# Functional and taxonomic dysbiosis of the supragingival plaque metagenome in Behçet’s disease

**DOI:** 10.1080/20002297.2025.2552165

**Published:** 2025-08-29

**Authors:** Thanyarat Sapthanakorn, Pitipol Choopong, Wasawat Sermsripong, Chatkoew Boriboonhirunsarn, Chompak Khamwachirapitak, Annop Krasaesin, Pimchanok Sutthiboonyapan, Nisachon Siripaiboonpong, Rangsini Mahanonda, Paswach Wiriyakijja, George Pelekos, Thantrira Porntaveetus, Supreda Suphanantachat Srithanyarat

**Affiliations:** a Department of Periodontology, Faculty of Dentistry, Chulalongkorn University, Bangkok, Thailand; b Department of Ophthalmology, Faculty of Medicine Siriraj Hospital, Mahidol University, Bangkok, Thailand; c Dental Department, Faculty of Medicine Siriraj Hospital, Mahidol University, Bangkok, Thailand; d Center of Excellence in Medicine and Digital Health, Faculty of Dentistry, Chulalongkorn University, Bangkok, Thailand; e Center of Excellence in Periodontal Disease and Dental Implant, Chulalongkorn University, Bangkok, Thailand; f Department of Oral Medicine, Faculty of Dentistry, Chulalongkorn University, Bangkok, Thailand; g Division of Periodontology and Implant Dentistry, Faculty of Dentistry, The University of Hong Kong, Hong Kong SAR, China; h Department of Physiology, Geriatric Dentistry and Special Patients Care International Program, Faculty of Dentistry, Chulalongkorn University, Bangkok, Thailand

**Keywords:** Immunity, oral microbiome, Behçet’s disease, spirochete, health disparity

## Abstract

**Background:**

Behçet’s Disease (BD), a complex autoinflammatory disorder, is increasingly linked to microbial dysbiosis, yet the specific microbial signatures and their functional consequences remain incompletely characterized. Elucidating these alterations is crucial for understanding BD pathogenesis.

**Objective:**

To identify distinct microbial community structures and functional potentials in supragingival plaque microbiomes of BD patients versus healthy controls (HC) using high-resolution shotgun metagenomic sequencing.

**Methods:**

Supragingival plaque from 18 BD patients and 22 HCs was subjected to shotgun metagenomics. Analyses included alpha/beta diversity, taxonomic composition, and MetaCyc pathway abundance, with statistical comparisons.

**Results:**

Despite similar age and clinical attachment levels, BD patients exhibited significantly increased alpha diversity and distinct beta diversity compared to HCs. Differential abundance analysis revealed an enrichment of anaerobic and opportunistic taxa in BD (implicating 4 phyla and 28 genera), alongside 19 significantly altered MetaCyc pathways, indicating substantial functional reprogramming within the BD oral microbiome.

**Conclusion:**

This high-resolution metagenomic analysis reveals profound oral microbiome dysbiosis in Behçet’s Disease, characterized by altered diversity, a distinct taxonomic signature enriched with pathobionts, and significant functional shifts. These comprehensive microbial alterations are implicated in contributing to the local and systemic inflammatory processes driving BD pathogenesis, offering potential avenues for diagnostic biomarkers and targeted therapies.

## Introduction

Behçet’s Disease (BD) is a multisystem autoinflammatory disorder characterized by oro-genital ulcers, cutaneous manifestations, and ocular, vascular, neurologic or gastrointestinal involvement [1]. BD affects people around the world with the global prevalence of 10.3:100,000 [2]. It is most prevalent along the Silk Road, a network of ancient trade routes connecting China to the Middle East and Europe [3], especially in Türkiye where the prevalence is almost 1:250 of the population above 12 years old [4–7]. Generally, it affects males and females equally, except in some Middle East and Mediterranean countries, where males predominate, and Japan and Korea, where on the contrary females are commonly affected [8]. These disparities may stem from an interplay of genetic, hormonal, immunological, and environmental influences. Differences in symptom presentation between males and females may also contribute to variations in disease recognition, reporting, and diagnosis across different populations. Greater healthcare access and awareness among women in some Asian countries may influence reporting rates. Genetic predisposition plays a key role in BD, with the Human Leukocyte Antigen (HLA)-B * 51 allele identified as the strongest associated risk factor [9]. Environmental factors such as viral or bacterial infections, consumption of histamine-releasing foods, poor oral hygiene, and psychological stress have been proposed as potential disease triggers [1]. Clinical manifestations of BD also differ by sex. Ocular involvement, vascular complications, and neurological symptoms are more frequently reported in male patients, whereas oral and genital ulcers, skin lesions, and arthritis are more common in female patients [10]. Moreover, disease activity tends to decline with age, with younger individuals and those with early-onset BD generally exhibit higher disease activity scores compared to older patients [11,12].

The primary symptom of BD is systemic vasculitis with involvement of multiple organs. Skin and mucocutaneous lesions are hallmark features of the disease. Other clinical manifestations may affect the ocular, cardiovascular, gastrointestinal, and central nervous systems. BD diagnosis is based on the screening criteria proposed by the International Study Group (ISG) [13], which require recurrent oral ulceration along with at least two additional features: recurrent genital ulcerations, characteristic eye lesions, skin lesions, or a positive pathergy test. Oral ulceration is defined as major, minor aphthous, or herpetiform ulcers occurring at least three times per year.

Due to the involvement of multiple organs, BD affects considerably on patient’s quality of life and some serious symptoms can be life-threatening [14,15]. Since its introduction in the 1930s, BD etiology and pathogenesis have not been fully explained [16]. Infectious microorganisms, however, have been one of the most studied factors, and molecular mimicry has been proposed as a mechanism that links infection to BD pathology. As some bacterial peptides contain similar peptide sequences to host protein, antibodies induced by those bacteria can become autoantibodies and cross-react with host protein. To our knowledge, no single microorganism or bacterial antigen has yet been identified as the etiology of BD.

Currently, the composition of the microbiome in BD patients and its role in the pathophysiology of BD have become a focus of research. Butyrate-producing bacterial species which generate short chain fatty acids play an important role in maintaining gut microbiome homeostasis. Several studies have reported a reduction in butyrate-producing species in the gut of patients with BD. Fecal samples from BD patients have shown a significant decrease in the alpha diversity of key butyrate-producing genera, including *Clostridium* spp., *Butyrivibrio* spp., and *Akkermansia* spp [17–19]. Butyrate contributes to immune regulation by suppressing intestinal pro-inflammatory cytokines and promoting the differentiation and function of regulatory T cells (Tregs). A decrease in butyrate levels may disrupt T-cell homeostasis, characterized by increased differentiation of pro-inflammatory T helper 17 (Th17) cells and reduced Treg populations. This imbalance has been hypothesized to impair immune regulation and contribute to gut inflammation in BD [17]. Additionally, the oral microbiome is well-documented to be associated with oral ulceration, the most common manifestation of BD [20]. However, studies on the composition of the oral microbiome in BD remain limited [21]. While research has reported varying results regarding the abundance or depletion of specific bacterial species due to the differences in study population or technique used, a common finding is reduced bacterial diversity in BD patients compared to those of the healthy controls [22–24]. This suggests that BD may alter the host’s oral bacterial community, potentially contributing to related disease manifestations such as bowel disease or oral ulceration [23–25].

Shotgun metagenomics is a powerful method for analyzing microbial communities without the need for prior culturing, offering significant advantages over traditional 16 S rRNA sequencing. While 16 S targets specific gene regions and is prone to PCR bias [26], shotgun metagenomics sequences all microbial DNA in a sample, enabling comprehensive identification of bacterial species [27,28]. This untargeted approach is especially valuable in oral microbiome studies, providing detailed taxonomic and functional profiles. By overcoming the limitations of targeted methods, it deepens our understanding of microbial diversity and interactions [29,30]. This study aims to compare the supragingival plaque microbiome of BD patients and non-BD controls using shotgun metagenomic sequencing to explore microbial shifts related to disease pathogenesis.

## Materials and methods

### Ethical approval

The ethics procedures were approved by the Human ethics research committee of Faculty of Dentistry, Chulalongkorn University (HREC-DCU 2022–090) and Faculty of Medicine Siriraj Hospital, Mahidol University (MU-MOU CoA No. 353/2023). Laboratory Biosafety was approved by the biosafety committee (CU-IBC 007–2023). All participants received study protocol, detailed information and signed informed consent was obtained.

### Participants

#### Sample size

This study is the first to investigate the oral microbiome in Behçet’s disease (BD) using shotgun metagenomic sequencing. The sample size was estimated using a formula for comparing two independent groups, based on data from Balt et al. [22]. Their study, which analyzed BD salivary microbiota using 16S rRNA sequencing, recommended a minimum of 21 participants per group. In a separate study, Wirth et al. [31] employed shotgun metagenomics to analyze the salivary microbiome in a similar context, including 22 participants (11 per group). The present study included 40 individuals (18 BD, 22 non-BD), exceeding the sample sizes of these prior studies and deemed sufficient for exploratory comparative analysis.

#### Inclusion criteria

Participants were recruited from the Department of Ophthalmology, Siriraj Hospital, Faculty of Medicine, Mahidol University and the Faculty of Dentistry, Chulalongkorn University. All subjects must be at least 18 years old with at least 20 natural teeth. Participants were divided into two groups: the Behçet’s Disease (BD) group and the healthy control (HC) group. BD patients met the International Study Group for Behçet’s Disease criteria (ISG criteria) [13]. The HC participants did not have BD.

#### Exclusion criteria

Participants were excluded if they had uncontrolled systemic diseases, such as cardiovascular diseases, diabetes, or obesity (defined as a BMI greater than 30 kg/m^2^). Other exclusion criteria included having oral candidiasis, being a current smoker, being pregnant, undergoing periodontal treatment within 3 months prior to sample collection, using antibiotics or antifungal drugs within 1 month prior to sample collection, or using antiseptic mouthwash products within 2 days prior to sample collection.

### Data and sample collection

#### Data collection

Demographic and medical history data were obtained from electronic medical records and interviews. Full mouth intraoral photographs were taken from five standard views. Periodontal conditions were assessed using a UNC-15 periodontal probe (Hu-Friedy, Chicago, USA) at six sites per tooth. Panoramic and/or intraoral radiographs were taken when necessary. Eye involvement was classified into anterior or posterior uveitis according to the International Uveitis Study Group criteria [32].

#### Sample collection

Participants were instructed to abstain from tooth brushing for 24 h prior to sample collection and to avoid consuming food and beverage for 2 h before the procedure. Supragingival plaque was collected from all remaining teeth as pooled supragingival plaque using a sterile Gracey curette or sickle (Hu-Friedy, Chicago, IL, USA). The samples were then transferred into separate Eppendorf tubes. After collection, the plaque samples were transported on an ice pack at −4°C and subsequently preserved at −80°C for further investigation [33].

#### DNA extraction, library preparation and sequencing

Bacterial microbiome DNA from supragingival samples was extracted using the QIAamp® DNA Microbiome Kit (Qiagen, Germany) [34] according to the manufacturer’s protocol. Initially, host nucleic acids from the sample were removed during incubation with Benzonase. Bacterial lysis was then performed using both mechanical and chemical methods, which included a detergent and large beads. Finally, pure bacterial DNA was eluted from the QIAamp UCP Mini Column using Buffer AVE. The concentration of the DNA was measured with the Thermo Scientific™ NanoDrop™ One Microvolume UV-Vis Spectrophotometer (Thermo Fisher Scientific, USA).

Microbial DNA samples that met the minimum quality standards were sent to Macrogen Inc. (Seoul, South Korea) for quality assessment before DNA sequencing. Paired-end whole-genome shotgun libraries were prepared from the genomic DNA using the Illumina DNA Library Prep Kit (Illumina, San Diego, CA, USA) following the manufacturer’s protocols. Paired-end sequencing was conducted using the NovaSeq X series (Illumina, San Diego, CA, USA). Subsequent quality control involved screening and removing raw reads with host contamination using KneadData (version 0.12), in conjunction with Trimmomatic (version 0.39) and Bowtie2 (version 2.5.1). Low-quality sequences were discarded, and the reads were mapped to the human genome (hg19, hg37 and human contamination reference database). Taxonomic profiling of the metagenomic shotgun data was performed using MetaPhlAn version 4.0.6 (The Huttenhower Lab, Boston, MA, USA). The oral microbiome profile data was then interpreted and analyzed using multivariate statistical methods. The functional profile of metagenomic shotgun data was performed by Humann3 version 3.6 [35]

#### Statistical analysis

Demographic data and clinical parameters were reported using descriptive statistics. The distribution of all demographic data and clinical parameters was tested for normality. The Chi-square test or Fisher’s Exact test was used to examine qualitative data. Differences in supragingival plaque microbiome data and functional profiles between BD patients and healthy controls were analyzed using the Mann-Whitney U test. Alpha diversity, based on the relative abundance in species levels, Shannon, Simpson, and Chao1 was calculated using the scikit-bio library version 0.6.0 in Python version 3.11.4. Beta diversity was assessed using a Bray-Curtis dissimilarity matrix for principal coordinate analysis (PCoA), and heatmaps were employed to examine the diversity between samples. A permutation multivariate analysis of variance (PERMANOVA) was conducted to compare beta diversity between BD patients and healthy controls with 999 permutations by Scikit-bio library version 0.6.0. A volcano plot was visualized between log_2_ fold change and false discovery rate (FDR) with Benjamini-Hochberg (BH) methods. A *P*-value of <0.05 was considered statistically significant. Statistical analysis was performed using the SPSS software package (version 22; SPSS Inc., Chicago, IL, USA) and SciPy library version 1.13.0. All illustrations were created using Matplotlib version 3.8.4 and Seaborn version 0.13.2 libraries in Python version 3.11.4.

### Results

#### Subject demographic data

**Figure 1.**
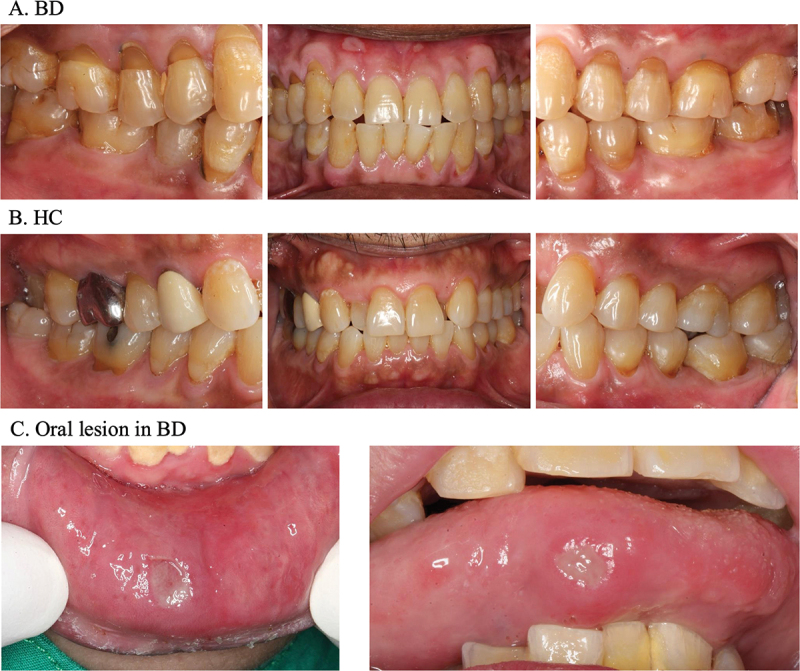
Clinical oral examination (A) 57-year-old man with Behçet’s disease, mean CAL 2.93 mm. (B) healthy 57-year-old man, mean CAL 2.03 mm. (C) oral lesion in Behçet’s disease; aphthous ulcers measuring 0.5–1.0 cm in diameter on the lower lip mucosa and the lateral border of the tongue. BD; Behçet group, HC; healthy control group.

The BD patient group comprised 16 males and 2 females, while the healthy control (HC) group included 11 males and 11 females, highlighting a significant male predominance (89%) in the BD group. The average age of BD patients was 42.49 ± 12.58 years, which was not significantly different from the mean age of the HC group (47.36 ± 14.57 years). There was no significant difference in the mean clinical attachment level between the BD and HC groups (2.51 mm vs. 2.52 mm). Among the 18 BD patients, 8 had evidence of eye involvement, including anterior or posterior uveitis, and 2 had oral ulcers at the time of sample collection (Figure 1(A–C)). Regarding medical treatment, all BD patients were on medication: 8 were treated with colchicine, 17 with immunosuppressants, and 3 with TNF inhibitors. Additionally, 10 BD patients were receiving a combination of two out of these three drug types (Table 1).

**Table 1.** Demographic data and clinical characteristics.

Characteristics	Total (*n* = 40)		BD (*n* = 18)		Non-BD (*n* = 22)		
	n	%	n	%	n	%	*P*-value
Gender							0.009*
Male	27	67.5%	16	88.9%	11	50.0%	
Female	13	32.5%	2	11.1%	11	50.0%	
Age (years)							
20–39	15	37.5%	7	38.9%	8	36.4%	
40–49	10	25.0%	6	33.3%	4	18.2%	
50–59	10	25.0%	4	22.2%	6	27.3%	
≥60	5	12.5%	1	5.6%	4	18.2%	
Mean±SD	45.17±13.76		42.49±12.58		47.36±14.57		0.270
Medication							
Colchicine			8	44.4%			
Immunosuppressant			17	94.4%			
TNF inhibitors			3	17.6%			
Eye involvement			8	44.4%			
Oral lesion			2	11.1%			
Mean CAL (mm)			2.51 (1.8–3.49)		2.52 (1.0–3.77)		

*P*-values for mean data were calculated with the use of Independent t-test, for percentages with the use of Chi-square test or Fisher’s exact test, * Significant at *P*-value < 0.05.

#### Composition of oral microbiota

**Figure 2.**
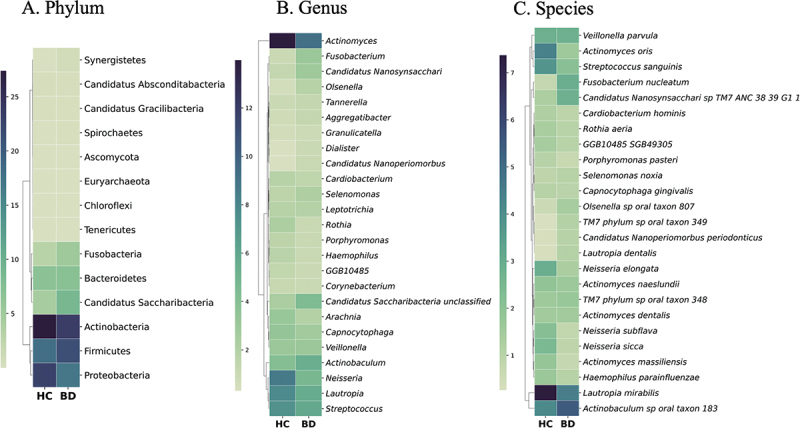
Heatmap displaying the top 25 taxa for 18 BD supragingival plaque samples compared to 22 HC samples. the phylum (A), genus (B), and species (C) levels. The color scale represents the average of relative abundance. BD; Behçet group, HC; healthy control group.

There were significant differences in the composition of phylum, genus, and species between the BD and HC groups (*P* < 0.05). To highlight these differences in taxonomic composition, a heatmap plot showed the top 25 by average of relative abundance at the phylum (Figure 2(A)), genus (Figure 2(B)), and species (Figure 2(C)) levels for each subject.

At the phylum level, Spirochaetes, Fusobacteria, Tenericutes, Candidatus, Saccharibacteria, Synergistetes, and Chloroflexi were notably more prevalent in the BD group (Supplementary Table S1). At the genus level, 28 genera showed significant differences between the BD and HC groups. *Candidatus absconditabacteria*, *Neisseria*, and GGB4936 genus (unclassified genus in Candidatus absconditabacteria phylum) were prevalent in the HC group. Other genera were prevalent in BD, such as *Campylobacter, Olsenella, Dialister, Fusobacterium, Shuttleworthia, Parvimonas, Fretibacterium, Megasphaera, Treponema, Solobacterium, Mogibacterium*, and *Slackia* (Supplementary Table S2).

At the species level, out of 479 detected species in supragingival plaque, there were 57 species that exhibited significant differences between the groups. Among these, the top five significantly abundant species in the BD group included *Fusobacterium nucleatum, Actinomyces oris, Neisseria elongata, Olsenella sp. oral taxon 807* and *Dialister invisus*. While, the top five dominant species in HC were *Actinomyces oris, Neisseria elongata, Fusobacterium nucleatum, Olsenella sp. oral taxon 807*, and *Actinomyces_SGB17163* (Supplementary Table S3).

#### Alpha and beta diversity

**Figure 3.**
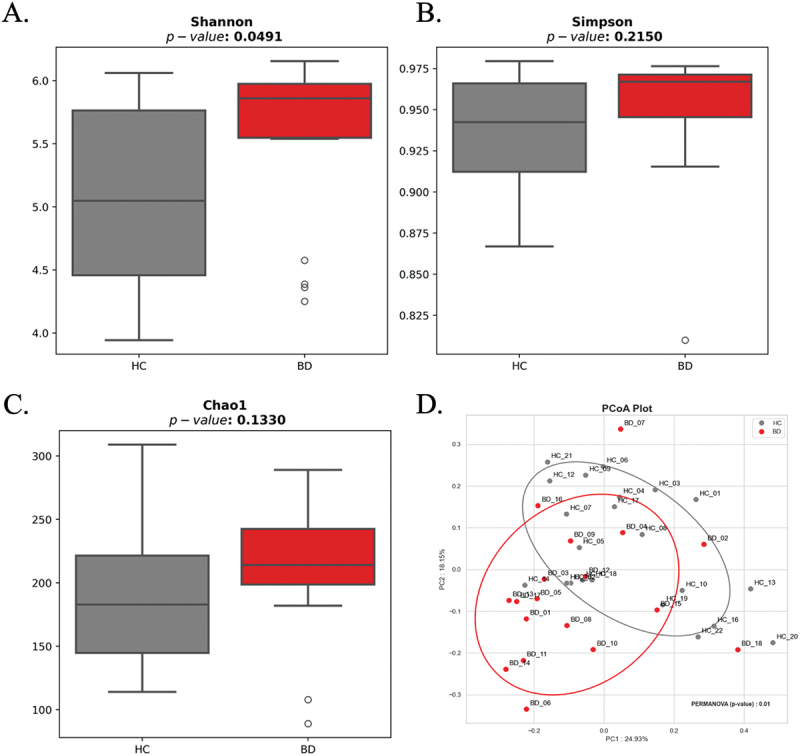
Comparison of alpha and beta diversity of supragingival microbiota between BC and HC groups. (A-C) boxplot of alpha-diversity shows significant in bacterial diversity between the BD and HC groups in Shanon Index, however, no difference in Simpson and Chao1. (D) beta-diversity presented through principal coordinates analysis (PCoA). The Bray-Curtis distances were utilized to indicate the percentage of variation explained in the microbial community. BD; Behçet group, HC; healthy control group.

The findings demonstrate a significant difference in alpha diversity between the BD and HC groups, specifically when assessed using the Shannon Diversity Index (*P*< 0.05). This suggests that individuals with Behçet’s disease exhibit higher overall bacterial diversity, capturing both species richness and evenness within the oral microbiome. In contrast, no significant differences were observed using Simpson’s Evenness Index and the Chao1 Richness Index, indicating that while overall diversity is elevated in BD, species evenness and richness alone are not markedly altered (Figure 3(A–C)).

Regarding beta diversity, principal coordinates analysis (PCoA) based on Bray-Curtis distances revealed a distinct clustering of the BD group compared to the HC group (PERMANOVA = 0.01), reflecting significant differences in microbial community composition (Figure 3(D)). These results collectively suggest that Behçet’s disease is associated with notable alterations in the structure and composition of the supragingival microbiota.

#### Functional analysis

**Figure 4.**
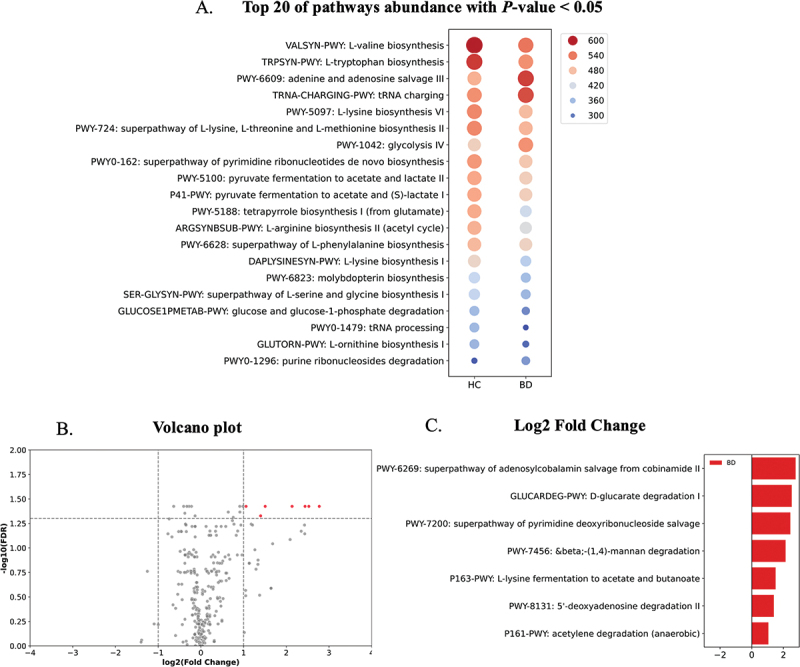
Functional and taxonomic differences in the oral microbiota between Behçet’s disease and healthy controls. (A) comparison of the top 20 significantly enriched pathways (*P* < 0.05) between the BD and HC groups. Pathways are ranked by enrichment strength and visualized using a color gradient. (B) volcano plot illustrating differentially abundant microbial species between BD and HC. The x-axis represents the log₂ Fold change, while the y-axis displays the – log₁₀ p-value. Statistically significant species are highlighted, revealing candidates potentially involved in BD pathogenesis. (C) log₂ Fold change of significantly enriched pathways (adjusted by FDR) identified in the BD group.BD: Behçet’s disease group; HC: healthy control group.

The top 20 significant abundance pathways were identified in both BD and HC groups (*P* < 0.05). Pathways are ranked by the strength of enrichment and reflected in color (Figure 4(A)). Pathways related to amino acid biosynthesis (e.g. valine, tryptophan, lysine) and nucleotide metabolism (e.g. adenine and adenosine salvage, pyrimidine biosynthesis) show higher abundance in the HC group. Conversely, several pathways, including those involved in glucose metabolism (glycolysis, gluconeogenesis), certain amino acid pathways (L-ornithine biosynthesis, L-serine and glycine biosynthesis), and heme biosynthesis tend to have higher abundance in the BD group. In contrast, pathways related to tRNA processing show higher abundance in the BD group (Figure 4(A)).

Volcano plots between FDR and log_2_ fold change significantly highlight pathways in BD patients compared to HC. The red dot represents pathways above the threshold. The volcano plot visualizes the -log_10_ FDR and log_2_ fold changes (Figure 4(B)). Significant pathways were selected by the volcano plot based on a fold-change threshold, which over 1 referred to as upregulated in BD and lower −1 referred to as downregulated in BD compared to HC groups. When comparing significant species by FDR between BD and HC groups, 19 pathways were significant abundance (FDR > 0.05). Among these, there were seven pathways which more prevalent in the BD group over two times, whereas no pathways showed significant prevalence in the HC. The super pathway of adenosylcobalamin salvage from cobinamide II pathway was the most significantly prevalent in the BD group, followed by the D-galactarate degradation I, super pathway of pyrimidine deoxyribonucleoside salvage, beta (1,4)-mannan degradation, L-lysin fermentation to acetate and butanoate, 5’-deoxyadenosine degradation II, and acetylene degradation (anaerobic) (Figure 4(C) and Supplementary Table S4). *Fusobacterium nucleatum* is a key species involved in L-lysine fermentation to acetate and butanoate, as well as in the acetylene degradation pathway. *Streptococcus anginosus* and *Streptococcus infantis* are significant species contributing to the acetylene degradation pathway. *Olsenella profusa* was found to be associated with the 5’-deoxyadenosine degradation II pathway (Supplementary Table S5).

## Discussion

The microbiome analysis in BD patients have previously been studied in various population including Turkish, British, Mongolian, and Dutch population [22–25]. Although those studies have identified microorganisms from salivary sample of BD patients, there is no conclusive evidence of these microorganisms linking directly to the disease [36]. The present study investigated the oral microbial composition in dental plaque of BD patients in a Thai population, using shotgun metagenomic analysis. Supragingival plaque is a useful diagnostic indicator and can be collected using a non-invasive technique. Supragingival plaque is a useful diagnostic indicator that can be collected using a non-invasive method. It forms a stable, structured biofilm with high microbial biomass in direct contact with the gingival margin. This microbial community is closely associated with periodontal inflammation, a well-studied model of chronic disease driven by persistent host – microbe interactions. Unlike saliva or mucosal swabs, which reflect more transient microbial populations, supragingival plaque offers a consistent microbial reservoir with established links to systemic inflammatory responses. Given the proposed association between oral microbial dysbiosis and BD, analysis of the plaque microbiome may provide valuable insights into potential oral contributions to systemic inflammation in BD.

Our results revealed a significant difference in bacterial composition at various taxonomic levels in supragingival plaque between BD and HC group. The BD patients exhibited elevated levels of gram-negative anaerobic species and opportunistic pathogens in their supragingival plaque, while gram-positive and facultative bacteria were less common. The previous study was also able to isolate anaerobic strains from the oral cavity in BD patients [37].

Our study demonstrates significant difference in alpha diversity between BD and HC group. The richness and evenness of microorganism in BD group were slightly higher than those in HC group. However, from the previous study using 16S rRNA V4 region technique, the diversity of salivary microbiome in BD group was less significant than that of HC group. The sensitivity of different technique may differentiate the result. Shotgun metagenomics offers a more complete and functional view of microbial communities as it can efficiently detect low abundant taxa [30]. This facilitated the in-depth information collection of the microbial community resided in supragingival plaque of BD patients.

Moreover, the diversity of microorganism in the HC group has wide range data, while BD group demonstrates similar data in each patient, obviously seen in the boxplot of alpha-diversity. We can imply that BD group have more homogeneous data since the disease activity in BD group was quite similar and all participants were undergoing treatment in our study. Of this study, 94% of BD group use immunsupressant drug, followed by colchicine and TNF-inhibitor for more than 5 years in order to suppress the eyes’ complication symtomps. While these medications are known to modulate inflammation, their long-term effects on the oral microbiome remain poorly understood. The diversity in BD’s oral microbiome might be influenced by their medication. Seoudi et al. [38] has reported 68% of BD patients undergoing topical and/or steroid treatment showed a decrease in *Neisseria* and *Veillonella* in salivary samples, with an increase in *Candida albicans*. Steroid treatment is recognized for elevating the risk of infection caused by *Candida spp*. Moreover, immunosuppressant drugs can alter salivary microbiome, potentially leading to the presence of opportunistic pathogens by limiting T-cell-mediated responses [39]. However, Ames et al. [40] noted that patients undergoing immunosuppressive therapy for severe aplastic anemia did not show a clear change in oral microbiome diversity over time, suggesting that the effects of such treatments may vary among individuals. The diversity of oral microbiome may vary due to the dynamic environment of the oral cavity, which is influenced by factors especially salivary flow [41].

Colchicine has anti-inflammatory effects but has been shown not to significantly alter the gut microbiome [42]. In BD patients, no significant differences were observed in the salivary microbiome between those treated with colchicine and those receiving systemic immunosuppressants. Although certain immunosuppressants can influence gut anaerobic bacterial population [43], and studies in spondyloarthritis, inflammatory bowel disease (IBD), and rheumatoid arthritis indicate that these therapies may modulate the microbiome [44], longitudinal investigations specifically assessing their effects on the oral microbiome remain lacking. This represents an important gap for future research.

The oral cavity and gut are linked both physically and chemically. Oral and oropharyngeal microbiota transfer to the gut through swallowed saliva, food, and drinks. If oral bacteria can endure the acidic conditions of the stomach, they may establish themselves and proliferate within the gastrointestinal tract. Their endotoxins can influence the gut microbiome, leading to potential alterations [45]. This disruption can affect the host immune response and further influence local inflammatory responses. Our BD patients’ supragingival plaque sample exhibited gram negative and facultative anaerobic bacteria. In BD group, the result also demonstrates the high abundance of *Tannerella forsynthia*, one of the red complex periodontal pathogen, whereas *Fusobacterium nucleatum* and *Campylobacter gracilis were in* the orange complex [46]. Previous studies have shown that BD patients have poorer periodontal conditions compared to HC patients. However, the severity of periodontal disease in these patients is generally mild. Reported mean values for BD patients include a Community Periodontal Index of Treatment Needs (CPITN) of 1.79–1.8, probing depth (PD) of 1.82–2.7 mm, and clinical attachment level (CAL) of 2.06–3.1 mm [47–52]. In the present study, although the BD group exhibited a higher abundance of virulence-associated bacteria – potentially indicating an increased risk of periodontal destruction – there was no significant difference in CAL between the BD and HC groups. This could be attributed to the fact that all BD patients in this study were receiving immunosuppressive therapy to manage their condition, which may also suppress the virulence of red and orange complex bacteria. Our findings suggest that maintaining good oral hygiene and adhering to regular supportive periodontal maintenance visits are essential for BD patients.

*Fusobacterium nucleatum* is common in the oral cavity but rare in the guts of healthy individuals. Interestingly, IBD patients showed colonization of *F. nucleatum* in the gut indicating the presence of the oral – gut microbiome axis in IBD patients [53]. Furthermore, the meta-analysis performed by She [54] has shown a strong association between periodontitis and two major forms of IBD: Crohn’s disease and ulcerative colitis.

Our study found representation of new species abundant in BD group such as *Campylobacter SGB19292, Campylobacter SGB 19,298, Olsenella sp. oral taxon 807, and Olsenella_SGB72635*. From previous studies, *Olsenella uli* has been reported in lung infection or pneumonia [55]. Moreover, there is the report showing the significant abundance of oral *Campylobacter spp*. involved with certain IBD or gastrointestinal ulcers [56], suggesting a potential link between oral and gut microbiome species. Although our participants’s GI tract or IBD condition have not been investigated, our results suggested that oral dysbiosis may involve in a modulation of pathogenesis in oral – gut axis.

Additionally identifying BD’s metabolic pathway alterations by functional analysis offers insights into the underlying mechanisms. *Fusobacterium nucleatum* plays an important role in amino acid and xenobiotic metabolism. In the L-lysine fermentation pathway, it ferments lysine into acetate and butanoate, both of which are short chain fatty acids known to influence host immune responses and maintain gut health. This metabolic flexibility allows *F. nucleatum* to adapt to anaerobic environments, contributing to microbial ecosystem dynamics and potentially to disease processes. While *F. nucleatum* participates in lysine fermentation within the gut, industrial microbial fermentation of L-lysine primarily involves strains such as *Escherichia coli*, *Corynebacterium glutamicum*, and *Brevibacterium flavum* [57].

*Olsenella profusa* is a Gram-positive anaerobic bacterium commonly found in the oral cavity and gastrointestinal tract. Increased activity of the 5’-deoxyadenosine degradation II pathway suggests a disturbance in purine metabolism, which is essential for immune cell growth and function. This pathway helps maintain nucleotide balance and removes inhibitory metabolic byproducts, supporting microbial growth and gut stability. The upregulation of this pathway in Behçet’s disease patients may contribute to immune system activation or reflect adaptive changes in microbial metabolism during disease. The superpathway of adenosylcobalamin salvage from cobinamide II is notably prevalent in BD, emphasizing the recycling of cobinamide to maintain adequate vitamin B12 levels. Disruptions in cobalamin metabolism may contribute to the inflammatory manifestations [58].

Microbiome-related pathways, particularly carbohydrate degradation and fermentation, are also implicated in BD. Mannan, a key component of the cellulose family, is classified into α- and β-mannan based on glycosidic linkages, both of which influence human gut microbiota composition and activity [59]. The prevalence of the β-(1,4)-mannan degradation pathway in BD may link to oral microbiota dysbiosis. Additionally, *Streptococcus anginosus* and *Streptococcus infantis* are facultative anaerobes grows by using acetylene degradation pathways, therefore it may indicate microbiome shifts and contribute to immune dysregulation in BD. This altered microbial energy metabolism could influence the overall metabolic state of BD patients, possibly contributing to systemic inflammation and tissue damage.

However, this study suffers from some limitations. Its cross-sectional design only allowed to determine the association between abundant bacterial communities and the disease. Moreover, the rarity of BD patients limited our study, contributing to the small sample size. This limitation may affect the generalizability of our findings and hinder the ability to draw robust conclusions about the microbial community in BD patients. Factors such as age, genetics, diet, stress, oral health, and medications can significantly impact the composition and changes in the bacterial community or microbiome [60]. Additionally, to prevent the severe complications such as blindness in BD, all participants were already prescribed different medications, which could have influenced the results.

Future studies should employ longitudinal research to focus on newly active BD cases and account for confounding factors. This approach will help better understand microbial shifts over time and enhance the accuracy and relevance of the findings. Additionally, using a larger sample size and inclusion of samples from the multiple oral niches, including the tongue and buccal mucosa, would be a valuable future direction that could reveal different aspects of microbial changes in this disease.

## Conclusion

Our study reveals significant alterations in the oral microbiome composition of supragingival plaque in BD patients, marked by an increased presence of gram-negative and opportunistic bacteria. Notably, Candidatus Saccharibacteria, Chloroflexi, Fusobacteria, Spirochaetes, Synergisterres, and Tenericutes phylum were found to be more prevalent in the BD group. Oral microbiome dysbiosis may be linked to the immune hyperreactivity a possible characteristic of the BD pathogenesis. This finding emphasizes the relevance of the oral and gut microbiomes in the disease’s development.

## Supplementary Material

01082025_R1_Supplementary_tablesclean.docx

## Data Availability

Metagenomic data were deposited to the National Center for Biotechnology Information (NCBI) by the sequence read archive (SRA), under BioProject accession number PRJNA1257016.
