# Supplementary material for: Functional and taxonomic dysbiosis of the supragingival plaque metagenome in Behçet’s disease
Source: J Oral Microbiol. 2025 Aug 29;17(1):2552165. doi: 10.1080/20002297.2025.2552165 (PMC12404063; doi:10.1080/20002297.2025.2552165)
Supplement: Supplementary file 1 — 01082025_R1_Supplementary_tablesclean.docx [file ZJOM_A_2552165_SM7887.docx]

**Supplementary Table 1. Phyla with significantly different relative abundances between BD and HC groups.**

| **Phylum** | **Average of HC** | **Average of BD** | **SD of HC** | **SD of BD** | ***P*-value** |
| --- | --- | --- | --- | --- | --- |
| Spirochaetes | 0.0762 | 0.2065 | 0.1103 | 0.1866 | 0.0080 |
| Fusobacteria | 2.9276 | 5.0671 | 1.8352 | 2.9276 | 0.0129 |
| Tenericutes | 0.0410 | 0.0931 | 0.1084 | 0.1247 | 0.0172 |
| Candidatus Saccharibacteria | 4.4994 | 8.9095 | 4.1864 | 5.8820 | 0.0174 |
| Synergistetes | 0.0892 | 0.7280 | 0.1316 | 1.1688 | 0.0206 |
| Chloroflexi | 0.0029 | 0.0896 | 0.0075 | 0.1834 | 0.0380 |

*P*-values for mean data were calculated with Mann-Whitney U test. SD: standard deviation. * Significant at *P*-value <0.05.

**Supplementary Table 2. Genus with significantly different relative abundances between BD and HC groups.**

| Genus | **Average of HC** | **Average of BD** | **SD of HC** | **SD of BD** | ***P*-value** |
| --- | --- | --- | --- | --- | --- |
| *GGB1202* | 0.0036 | 0.0547 | 0.0050 | 0.1416 | 0.0002 |
| *Shuttleworthia* | 0.0051 | 0.0380 | 0.0103 | 0.0483 | 0.0018 |
| *Olsenella* | 0.5989 | 1.8706 | 0.7866 | 1.5241 | 0.0036 |
| *Fusobacterium* | 1.0209 | 3.0350 | 0.8700 | 2.7683 | 0.0041 |
| *Dialister* | 0.3129 | 1.0240 | 0.3457 | 0.8996 | 0.0049 |
| *Candidatus Absconditabacteria unclassified* | 0.1532 | 0.2395 | 0.3918 | 0.2130 | 0.0050 |
| *Campylobacter* | 0.1600 | 0.6490 | 0.1399 | 0.6246 | 0.0058 |
| *Fretibacterium* | 0.0286 | 0.1750 | 0.0637 | 0.2320 | 0.0074 |
| *Treponema* | 0.0760 | 0.2034 | 0.1102 | 0.1802 | 0.0080 |
| *Megasphaera* | 0.0285 | 0.1002 | 0.0474 | 0.1267 | 0.0082 |
| *Solobacterium* | 0.0769 | 0.2268 | 0.1049 | 0.2380 | 0.0110 |
| *Slackia* | 0.0162 | 0.0717 | 0.0250 | 0.0991 | 0.0121 |
| *Anaeroglobus* | 0.1940 | 0.5565 | 0.3626 | 0.8449 | 0.0186 |
| *Neisseria* | 8.6547 | 4.7896 | 5.8577 | 4.1419 | 0.0187 |
| *GGB4733* | 0.0406 | 0.0928 | 0.1074 | 0.1248 | 0.0201 |
| *GGB12761* | 0.0085 | 0.0608 | 0.0389 | 0.1151 | 0.0204 |
| *GGB4533* | 0.1012 | 0.2535 | 0.2885 | 0.5416 | 0.0221 |
| *Mogibacterium* | 0.0435 | 0.1694 | 0.0688 | 0.2231 | 0.0231 |
| *Hallella* | 0.0000 | 0.0006 | 0.0000 | 0.0017 | 0.0231 |
| *Lactobacillus* | 0.0000 | 0.0004 | 0.0000 | 0.0011 | 0.0231 |
| *Pyramidobacter* | 0.0000 | 0.0461 | 0.0000 | 0.1774 | 0.0231 |
| *Atopobium* | 0.0026 | 0.0682 | 0.0104 | 0.1757 | 0.0232 |
| *Anaerolineaceae unclassified* | 0.0029 | 0.0896 | 0.0075 | 0.1834 | 0.0380 |
| *GGB4936* | 0.0576 | 0.0063 | 0.1323 | 0.0178 | 0.0380 |
| *Cryptobacterium* | 0.0062 | 0.0840 | 0.0170 | 0.2235 | 0.0441 |
| *Candidatus Saccharibacteria unclassified* | 2.4039 | 4.3810 | 3.2298 | 3.6520 | 0.0487 |
| *Parvimonas* | 0.1210 | 0.8390 | 0.1188 | 1.1165 | 0.0487 |
| *Eggerthia* | 0.0007 | 0.0562 | 0.0031 | 0.2011 | 0.0494 |

*P*-values for mean data were calculated with Mann-Whitney U test. SD: standard deviation. * Significant at *P*-value <0.05.

**Supplementary Table 3. Species with significantly different relative abundances between BD and HC group (Bold text: top 5 of dominant species in each group)**

| **Species** | **Average of HC** | **Average of BD** | **SD of HC** | **SD of BD** | **P-value** |
| --- | --- | --- | --- | --- | --- |
| *Actinomyces oris* | **4.3813** | **1.5933** | 5.3178 | 2.1252 | 0.0267 |
| *Neisseria elongata* | **2.9303** | **1.3688** | 2.0538 | 1.3073 | 0.0063 |
| *Fusobacterium nucleatum* | **0.8040** | **2.9401** | 0.7593 | 2.7627 | 0.0007 |
| *Olsenella sp oral taxon 807* | **0.5722** | **1.4186** | 0.7719 | 1.0427 | 0.0053 |
| *Actinomyces SGB17163* | **0.4933** | 0.0985 | 0.5762 | 0.1116 | 0.0149 |
| *Dialister invisus* | 0.2978 | **0.9501** | 0.3266 | 0.8380 | 0.0068 |
| *Anaeroglobus geminatus* | 0.1940 | 0.5565 | 0.3626 | 0.8449 | 0.0186 |
| *Streptococcus infantis* | 0.1522 | 0.0606 | 0.2699 | 0.0932 | 0.0487 |
| *SR1 bacterium human oral taxon HOT 345* | 0.1516 | 0.1846 | 0.3921 | 0.1967 | 0.0163 |
| *Oribacterium sp oral taxon 078* | 0.1059 | 0.2647 | 0.0915 | 0.2291 | 0.0400 |
| *Eubacterium infirmum* | 0.1049 | 0.3039 | 0.1768 | 0.3979 | 0.0417 |
| *Streptococcus anginosus* | 0.0945 | 0.3168 | 0.1468 | 0.5035 | 0.0319 |
| *Granulicatella SGB8255* | 0.0828 | 0.0201 | 0.2196 | 0.0267 | 0.0286 |
| *Solobacterium moorei* | 0.0741 | 0.2192 | 0.1045 | 0.2372 | 0.0231 |
| *Selenomonas sputigena* | 0.0721 | 0.2378 | 0.0967 | 0.2199 | 0.0073 |
| *GGB4936 SGB6889* | 0.0576 | 0.0063 | 0.1323 | 0.0178 | 0.0380 |
| *Treponema socranskii* | 0.0463 | 0.0994 | 0.0782 | 0.0863 | 0.0114 |
| *GGB4733 SGB6557* | 0.0406 | 0.0928 | 0.1074 | 0.1248 | 0.0201 |
| *GGB4533 SGB6246* | 0.0403 | 0.2241 | 0.1089 | 0.5443 | 0.0163 |
| *Prevotella nanceiensis* | 0.0383 | 0.0051 | 0.1163 | 0.0130 | 0.0488 |
| *Prevotella maculosa* | 0.0328 | 0.0660 | 0.0443 | 0.0770 | 0.0455 |
| *Fretibacterium fastidiosum* | 0.0286 | 0.1750 | 0.0637 | 0.2320 | 0.0074 |
| *Megasphaera micronuciformis* | 0.0285 | 0.1002 | 0.0474 | 0.1267 | 0.0082 |
| *Lachnoanaerobaculum sp ICM7* | 0.0283 | 0.0057 | 0.0508 | 0.0138 | 0.0284 |
| *Mogibacterium diversum* | 0.0258 | 0.0403 | 0.0566 | 0.0357 | 0.0062 |
| *Capnocytophaga SGB78737* | 0.0242 | 0.0000 | 0.0807 | 0.0000 | 0.0054 |
| *Campylobacter SGB19292* | 0.0230 | 0.0814 | 0.0255 | 0.0597 | 0.0010 |
| *Campylobacter SGB19298* | 0.0226 | 0.1106 | 0.0308 | 0.1226 | 0.0035 |
| *Prevotella denticola* | 0.0209 | 0.2747 | 0.0519 | 0.8381 | 0.0228 |
| *Olsenella uli* | 0.0207 | 0.2833 | 0.0306 | 0.6047 | 0.0302 |
| *Slackia exigua* | 0.0162 | 0.0717 | 0.0250 | 0.0991 | 0.0121 |
| *Campylobacter gracilis* | 0.0157 | 0.0469 | 0.0219 | 0.0441 | 0.0038 |
| *Dialister pneumosintes* | 0.0151 | 0.0736 | 0.0287 | 0.1094 | 0.0343 |
| *Selenomonas flueggei* | 0.0143 | 0.0852 | 0.0295 | 0.1700 | 0.0096 |
| *Campylobacter curvus* | 0.0136 | 0.0719 | 0.0339 | 0.1157 | 0.0381 |
| *Tannerella forsythia* | 0.0134 | 0.2359 | 0.0263 | 0.4499 | 0.0489 |
| *Gemella haemolysans* | 0.0116 | 0.0140 | 0.0268 | 0.0165 | 0.0259 |
| *GGB12761 SGB19791* | 0.0085 | 0.0608 | 0.0389 | 0.1151 | 0.0204 |
| *Streptococcus rubneri* | 0.0078 | 0.0005 | 0.0174 | 0.0012 | 0.0437 |
| *Prevotella micans* | 0.0063 | 0.0146 | 0.0129 | 0.0207 | 0.0427 |
| *Cryptobacterium curtum* | 0.0062 | 0.0840 | 0.0170 | 0.2235 | 0.0441 |
| *Shuttleworthia satelles* | 0.0051 | 0.0380 | 0.0103 | 0.0483 | 0.0018 |
| *Catonella SGB69305* | 0.0036 | 0.0077 | 0.0120 | 0.0090 | 0.0192 |
| *GGB1202 SGB1566* | 0.0036 | 0.0546 | 0.0050 | 0.1416 | 0.0002 |
| *Olsenella SGB72635* | 0.0032 | 0.0317 | 0.0119 | 0.0739 | 0.0308 |
| *Olsenella profusa* | 0.0029 | 0.0884 | 0.0096 | 0.3381 | 0.0192 |
| *Anaerolineaceae bacterium oral taxon 439* | 0.0029 | 0.0896 | 0.0075 | 0.1834 | 0.0380 |
| *Prevotella enoeca* | 0.0028 | 0.0057 | 0.0088 | 0.0090 | 0.0225 |
| *Candidatus Gracilibacteria bacterium HOT 871* | 0.0026 | 0.0392 | 0.0118 | 0.1285 | 0.0489 |
| *Prevotella baroniae* | 0.0023 | 0.0173 | 0.0062 | 0.0228 | 0.0165 |
| *Treponema SGB69443* | 0.0007 | 0.0037 | 0.0022 | 0.0073 | 0.0372 |
| *Eggerthia catenaformis* | 0.0007 | 0.0562 | 0.0031 | 0.2011 | 0.0494 |
| *Prevotella koreensis* | 0.0003 | 0.0076 | 0.0011 | 0.0184 | 0.0494 |
| *Prevotella buccae* | 0.0000 | 0.0021 | 0.0001 | 0.0043 | 0.0146 |
| *Pyramidobacter piscolens* | 0.0000 | 0.0461 | 0.0000 | 0.1774 | 0.0231 |
| *Parvimonas sp oral taxon 393* | 0.0000 | 0.0028 | 0.0000 | 0.0075 | 0.0231 |
| *Hallella seregens* | 0.0000 | 0.0006 | 0.0000 | 0.0017 | 0.0231 |

*P*-values for mean data were calculated with Mann-Whitney U test. SD: standard deviation. * Significant at *P*-value <0.05.

**Supplementary Table 4. Pathway with significantly different relative abundance between BD and HC group**

| Pathway | Average HC | Average BD | SD.of HC | SD of BD | *P*-value | log2fc | FDR |
| --- | --- | --- | --- | --- | --- | --- | --- |
| PWY-7456: &beta;-(1,4)-mannan degradation | 2.5502 | 11.1999 | 3.2469 | 17.8085 | 0.0002 | 2.1348 | 0.0376 |
| GLUCARDEG-PWY: D-glucarate degradation I | 0.3549 | 2.0462 | 0.8977 | 2.6515 | 0.0005 | 2.5275 | 0.0376 |
| PWY-5188: tetrapyrrole biosynthesis I (from glutamate) | 500.6047 | 412.8384 | 60.7772 | 63.0841 | 0.0005 | -0.2781 | 0.0376 |
| DAPLYSINESYN-PWY: L-lysine biosynthesis I | 450.5093 | 386.7516 | 40.7298 | 57.3805 | 0.0006 | -0.2201 | 0.0376 |
| P163-PWY: L-lysine fermentation to acetate and butanoate | 16.3756 | 46.5098 | 14.6643 | 35.3928 | 0.0007 | 1.5060 | 0.0376 |
| PWY-6507: 4-deoxy-L-threo-hex-4-enopyranuronate degradation | 13.2160 | 24.8461 | 6.2821 | 11.2809 | 0.0007 | 0.9107 | 0.0376 |
| GLUTORN-PWY: L-ornithine biosynthesis I | 353.1945 | 281.3998 | 62.3944 | 54.0241 | 0.0011 | -0.3278 | 0.0376 |
| PWY-7200: superpathway of pyrimidine deoxyribonucleoside salvage | 4.6264 | 25.0832 | 6.0105 | 32.2986 | 0.0012 | 2.4388 | 0.0376 |
| PWY4LZ-257: superpathway of fermentation (Chlamydomonas reinhardtii) | 26.1509 | 51.7435 | 14.9932 | 27.1047 | 0.0013 | 0.9845 | 0.0376 |
| PWY-6269: superpathway of adenosylcobalamin salvage from cobinamide II | 0.5883 | 4.0185 | 1.3609 | 5.2089 | 0.0014 | 2.7720 | 0.0376 |
| SO4ASSIM-PWY: assimilatory sulfate reduction I | 203.6024 | 130.9524 | 64.8488 | 57.2828 | 0.0014 | -0.6367 | 0.0376 |
| P161-PWY: acetylene degradation (anaerobic) | 34.6299 | 72.0207 | 20.2598 | 37.9696 | 0.0015 | 1.0564 | 0.0376 |
| PWY-5918: superpathway of heme b biosynthesis from glutamate | 303.6523 | 229.6517 | 65.9819 | 56.3351 | 0.0015 | -0.4030 | 0.0376 |
| PWY-6588: pyruvate fermentation to acetone | 29.2151 | 55.0456 | 19.1101 | 26.7116 | 0.0020 | 0.9139 | 0.0431 |
| PWY-7392: taxadiene biosynthesis (engineered) | 118.5529 | 184.7531 | 53.8687 | 63.5708 | 0.0020 | 0.6401 | 0.0431 |
| PWY-5920: superpathway of heme b biosynthesis from glycine | 157.4598 | 121.2822 | 43.1493 | 29.3030 | 0.0022 | -0.3766 | 0.0442 |
| PWY-8131: 5'-deoxyadenosine degradation II | 10.1902 | 26.8606 | 8.2763 | 22.2028 | 0.0027 | 1.3983 | 0.0471 |
| PWY0-162: superpathway of pyrimidine ribonucleotides de novo biosynthesis | 517.7289 | 471.2632 | 38.3100 | 62.2662 | 0.0027 | -0.1357 | 0.0471 |
| PWY-8190: L-glutamate degradation XI (reductive Stickland reaction) | 8.8560 | 14.9781 | 7.7644 | 4.7188 | 0.0029 | 0.7581 | 0.0487 |
| HEME-BIOSYNTHESIS-II-1: heme b biosynthesis V (aerobic) | 226.4848 | 135.2786 | 93.1231 | 81.5947 | 0.0032 | -0.7435 | 0.0506 |
| PWY-6549: L-glutamine biosynthesis III | 31.1720 | 52.0145 | 14.9997 | 21.1558 | 0.0035 | 0.7387 | 0.0526 |
| PWY-5690: TCA cycle II (plants and fungi) | 23.1126 | 39.5308 | 11.9772 | 17.9052 | 0.0041 | 0.7743 | 0.0572 |
| GLYCOL-GLYOXDEG-PWY: superpathway of glycol metabolism and degradation | 2.1294 | 4.8199 | 4.6059 | 4.5503 | 0.0042 | 1.1786 | 0.0572 |
| HEMESYN2-PWY: heme b biosynthesis II (oxygen-independent) | 312.6519 | 244.4897 | 70.9393 | 66.0669 | 0.0045 | -0.3548 | 0.0572 |
| P4-PWY: superpathway of L-lysine, L-threonine and L-methionine biosynthesis I | 282.3447 | 219.7139 | 54.6968 | 61.5793 | 0.0045 | -0.3618 | 0.0572 |
| PWY-5189: tetrapyrrole biosynthesis II (from glycine) | 282.9534 | 222.1549 | 59.6800 | 54.4581 | 0.0049 | -0.3490 | 0.0584 |
| DARABCATK12-PWY: D-arabinose degradation I | 0.7271 | 3.9083 | 1.0582 | 4.5567 | 0.0053 | 2.4263 | 0.0584 |
| POLYAMINSYN3-PWY: superpathway of polyamine biosynthesis II | 9.6934 | 22.3929 | 8.5069 | 14.8560 | 0.0053 | 1.2080 | 0.0584 |
| PWY-5676: acetyl-CoA fermentation to butanoate II | 32.9941 | 63.1517 | 24.3914 | 36.6720 | 0.0053 | 0.9366 | 0.0584 |
| POLYAMSYN-PWY: superpathway of polyamine biosynthesis I | 49.5921 | 74.2419 | 23.3776 | 24.5659 | 0.0058 | 0.5821 | 0.0603 |
| THISYNARA-PWY: superpathway of thiamine diphosphate biosynthesis III (eukaryotes) | 150.0705 | 192.3798 | 39.8141 | 46.4892 | 0.0063 | 0.3583 | 0.0603 |
| TRNA-CHARGING-PWY: tRNA charging | 524.8907 | 570.9017 | 55.0825 | 32.3094 | 0.0063 | 0.1212 | 0.0603 |
| COLANSYN-PWY: colanic acid building blocks biosynthesis | 103.8606 | 144.4024 | 44.4313 | 39.3439 | 0.0068 | 0.4754 | 0.0603 |
| P41-PWY: pyruvate fermentation to acetate and (S)-lactate I | 505.3099 | 460.1516 | 44.6662 | 58.7747 | 0.0068 | -0.1351 | 0.0603 |
| PWY-5100: pyruvate fermentation to acetate and lactate II | 505.3099 | 460.1516 | 44.6662 | 58.7747 | 0.0068 | -0.1351 | 0.0603 |
| ARGSYNBSUB-PWY: L-arginine biosynthesis II (acetyl cycle) | 494.1619 | 427.9537 | 70.4538 | 56.7724 | 0.0068 | -0.2075 | 0.0603 |
| PWY-7992: superpathway of menaquinol-8 biosynthesis III | 21.1501 | 38.4339 | 15.5998 | 18.9458 | 0.0080 | 0.8617 | 0.0672 |
| PWY-5659: GDP-mannose biosynthesis | 177.6422 | 205.7506 | 64.1396 | 33.1688 | 0.0080 | 0.2119 | 0.0672 |
| PWY-6749: CMP-legionaminate biosynthesis I | 0.2591 | 1.3205 | 0.5077 | 2.0462 | 0.0085 | 2.3496 | 0.0675 |
| BIOTIN-BIOSYNTHESIS-PWY: biotin biosynthesis I | 118.4637 | 150.1958 | 80.8726 | 38.0375 | 0.0087 | 0.3424 | 0.0675 |
| PWY0-1479: tRNA processing | 356.6680 | 258.3988 | 120.7048 | 108.4038 | 0.0087 | -0.4650 | 0.0675 |
| SULFATE-CYS-PWY: superpathway of sulfate assimilation and cysteine biosynthesis | 235.6637 | 185.9692 | 57.6362 | 51.5972 | 0.0094 | -0.3417 | 0.0681 |
| PWY0-1061: superpathway of L-alanine biosynthesis | 275.7931 | 201.0093 | 91.5729 | 75.5409 | 0.0094 | -0.4563 | 0.0681 |
| HEME-BIOSYNTHESIS-II: heme b biosynthesis I (aerobic) | 244.0337 | 169.9535 | 83.2157 | 68.2311 | 0.0094 | -0.5219 | 0.0681 |
| GLYOXYLATE-BYPASS: glyoxylate cycle | 104.0163 | 60.9522 | 65.1994 | 44.2356 | 0.0102 | -0.7711 | 0.0717 |
| PWY-7031: protein N-glycosylation (bacterial) | 0.3064 | 1.6532 | 0.5610 | 2.0400 | 0.0104 | 2.4319 | 0.0717 |
| PWY-6519: 8-amino-7-oxononanoate biosynthesis I | 111.2145 | 143.0049 | 85.2842 | 41.6539 | 0.0110 | 0.3627 | 0.0743 |
| PWY-7371: 1,4-dihydroxy-6-naphthoate biosynthesis II | 23.2910 | 41.3294 | 21.4871 | 21.0238 | 0.0119 | 0.8274 | 0.0743 |
| PWY-7199: pyrimidine deoxyribonucleosides salvage | 271.5244 | 319.2711 | 62.4936 | 41.1231 | 0.0119 | 0.2337 | 0.0743 |
| PWY-6609: adenine and adenosine salvage III | 495.4414 | 578.2846 | 99.7488 | 90.3578 | 0.0119 | 0.2231 | 0.0743 |
| PWY-1042: glycolysis IV | 459.1754 | 523.3045 | 89.1946 | 59.1243 | 0.0119 | 0.1886 | 0.0743 |
| PWY-5097: L-lysine biosynthesis VI | 531.0015 | 483.9814 | 43.6121 | 65.6787 | 0.0129 | -0.1338 | 0.0772 |
| PWY-5855: ubiquinol-7 biosynthesis (early decarboxylation) | 113.0527 | 70.6381 | 61.9502 | 50.4459 | 0.0129 | -0.6785 | 0.0772 |
| PWY-6748: nitrate reduction VII (denitrification) | 0.5667 | 2.4108 | 1.0324 | 2.8137 | 0.0139 | 2.0888 | 0.0821 |
| GALACTUROCAT-PWY: D-galacturonate degradation I | 0.7526 | 2.4111 | 1.2617 | 3.8870 | 0.0157 | 1.6798 | 0.0906 |
| SER-GLYSYN-PWY: superpathway of L-serine and glycine biosynthesis I | 399.9597 | 355.8061 | 56.7369 | 41.1505 | 0.0187 | -0.1688 | 0.1043 |
| PWY-7858: (5Z)-dodecenoate biosynthesis II | 246.3292 | 188.6716 | 90.5528 | 81.3567 | 0.0187 | -0.3847 | 0.1043 |
| PWY-5514: UDP-N-acetyl-D-galactosamine biosynthesis II | 0.6497 | 1.5604 | 1.3838 | 1.5101 | 0.0190 | 1.2640 | 0.1044 |
| PWY-7883: anhydromuropeptides recycling II | 186.5836 | 133.2808 | 74.3128 | 64.5508 | 0.0201 | -0.4854 | 0.1083 |
| PWY-5910: superpathway of geranylgeranyldiphosphate biosynthesis I (via mevalonate) | 66.0107 | 87.7172 | 25.9936 | 26.4971 | 0.0216 | 0.4102 | 0.1108 |
| PWY-724: superpathway of L-lysine, L-threonine and L-methionine biosynthesis II | 529.9527 | 490.1636 | 45.4146 | 59.7068 | 0.0216 | -0.1126 | 0.1108 |
| PWY-7115: C4 photosynthetic carbon assimilation cycle, NAD-ME type | 191.7482 | 158.7478 | 45.9646 | 32.1524 | 0.0216 | -0.2725 | 0.1108 |
| PWY-5347: superpathway of L-methionine biosynthesis (transsulfuration) | 251.7334 | 212.6205 | 58.2626 | 60.0584 | 0.0232 | -0.2436 | 0.1153 |
| PWY0-781: aspartate superpathway | 263.0291 | 212.8521 | 60.4405 | 58.5385 | 0.0232 | -0.3054 | 0.1153 |
| PWY0-1296: purine ribonucleosides degradation | 266.7814 | 324.6829 | 65.1476 | 77.3232 | 0.0249 | 0.2834 | 0.1180 |
| PWY-7118: chitin deacetylation | 7.6768 | 14.9652 | 10.3544 | 12.4117 | 0.0256 | 0.9630 | 0.1180 |
| PWY-7323: superpathway of GDP-mannose-derived O-antigen building blocks biosynthesis | 23.6396 | 42.7040 | 17.4459 | 25.6496 | 0.0267 | 0.8532 | 0.1180 |
| PWY-622: starch biosynthesis | 121.9502 | 191.1315 | 91.6060 | 87.9723 | 0.0267 | 0.6483 | 0.1180 |
| GLUCOSE1PMETAB-PWY: glucose and glucose-1-phosphate degradation | 359.8075 | 312.0383 | 69.2176 | 57.1572 | 0.0267 | -0.2055 | 0.1180 |
| HOMOSER-METSYN-PWY: L-methionine biosynthesis I | 205.1711 | 160.7630 | 61.0477 | 60.4797 | 0.0267 | -0.3519 | 0.1180 |
| NAGLIPASYN-PWY: lipid IVA biosynthesis (E. coli) | 243.0721 | 188.0068 | 81.6541 | 69.4614 | 0.0267 | -0.3706 | 0.1180 |
| PWY-8073: lipid IVA biosynthesis (P. putida) | 243.0721 | 188.0068 | 81.6541 | 69.4614 | 0.0267 | -0.3706 | 0.1180 |
| PWY-5861: superpathway of demethylmenaquinol-8 biosynthesis I | 33.2371 | 49.2209 | 15.8608 | 25.1205 | 0.0307 | 0.5665 | 0.1235 |
| PWY-6305: superpathway of putrescine biosynthesis | 53.7105 | 67.6107 | 18.4972 | 15.2036 | 0.0307 | 0.3320 | 0.1235 |
| PWY0-1241: ADP-L-glycero-&beta;-D-manno-heptose biosynthesis | 83.8373 | 99.1444 | 44.4599 | 26.2985 | 0.0307 | 0.2419 | 0.1235 |
| PWY-6628: superpathway of L-phenylalanine biosynthesis | 485.6812 | 455.7180 | 43.4512 | 39.7940 | 0.0307 | -0.0919 | 0.1235 |
| VALSYN-PWY: L-valine biosynthesis | 602.3639 | 543.9046 | 65.3732 | 69.8282 | 0.0307 | -0.1473 | 0.1235 |
| TRPSYN-PWY: L-tryptophan biosynthesis | 581.5562 | 524.8502 | 66.6629 | 76.2062 | 0.0307 | -0.1480 | 0.1235 |
| METSYN-PWY: superpathway of L-homoserine and L-methionine biosynthesis | 281.1656 | 228.9069 | 71.0894 | 72.5892 | 0.0307 | -0.2967 | 0.1235 |
| P124-PWY: Bifidobacterium shunt | 43.8897 | 70.2368 | 37.3450 | 44.9196 | 0.0328 | 0.6783 | 0.1305 |
| PWY-5464: superpathway of cytosolic glycolysis (plants), pyruvate dehydrogenase and TCA cycle | 3.6580 | 9.2491 | 6.2564 | 10.0344 | 0.0339 | 1.3383 | 0.1331 |
| PWY-5838: superpathway of menaquinol-8 biosynthesis I | 47.6288 | 67.9021 | 22.0074 | 31.7241 | 0.0351 | 0.5116 | 0.1362 |
| P122-PWY: heterolactic fermentation | 49.4506 | 64.8163 | 30.8309 | 24.0368 | 0.0375 | 0.3904 | 0.1421 |
| PWY0-1298: superpathway of pyrimidine deoxyribonucleosides degradation | 102.4714 | 126.0154 | 29.4637 | 33.6270 | 0.0375 | 0.2984 | 0.1421 |
| PWY-7384: anaerobic energy metabolism (invertebrates, mitochondrial) | 1.9133 | 4.2161 | 3.7311 | 5.3837 | 0.0391 | 1.1399 | 0.1433 |
| PWY-7389: superpathway of anaerobic energy metabolism (invertebrates) | 3.0335 | 6.6401 | 5.8565 | 8.3957 | 0.0391 | 1.1302 | 0.1433 |
| PWY-5005: biotin biosynthesis II | 37.2643 | 52.2129 | 22.6425 | 24.9675 | 0.0401 | 0.4866 | 0.1433 |
| HISDEG-PWY: L-histidine degradation I | 89.5201 | 121.4092 | 30.7125 | 45.9666 | 0.0401 | 0.4396 | 0.1433 |
| PWY0-1261: anhydromuropeptides recycling I | 247.6837 | 190.8461 | 90.2392 | 75.2862 | 0.0401 | -0.3761 | 0.1433 |
| GLYCOLYSIS-TCA-GLYOX-BYPASS: superpathway of glycolysis, pyruvate dehydrogenase, TCA, and glyoxylate bypass | 3.5160 | 8.8740 | 6.1867 | 10.3755 | 0.0416 | 1.3356 | 0.1471 |
| PWY-7211: superpathway of pyrimidine deoxyribonucleotides de novo biosynthesis | 9.9077 | 24.2443 | 13.0187 | 25.3647 | 0.0487 | 1.2910 | 0.1648 |
| PWY-6859: all-trans-farnesol biosynthesis | 103.2021 | 128.0473 | 39.9041 | 36.9946 | 0.0487 | 0.3112 | 0.1648 |
| GLUCONEO-PWY: gluconeogenesis I | 258.6301 | 279.9997 | 54.5621 | 27.1101 | 0.0487 | 0.1145 | 0.1648 |
| PWY-6823: molybdopterin biosynthesis | 403.8342 | 361.8977 | 81.1923 | 68.5993 | 0.0487 | -0.1582 | 0.1648 |

*P*-values for mean data were calculated with Mann-Whitney U test.

S.D.: standard deviation.

FDR: false discovery rate with Benjamini-Hochberg methods.

* Significant at *P*-value <0.05.

**Supplementary Table 5. Associated species with significant pathways**

| **Pathways Code** | **Name** | **Associatred species** |
| --- | --- | --- |
| PWY-6269 | superpathway of adenosylcobalamin salvage from cobinamide II | No specific species |
| GLUCARDEG-PWY | D-glucarate degradation I | No specific species |
| PWY-7200 | superpathway of pyrimidine deoxyribonucleoside salvage | No specific species |
| PWY-7456 | &beta;-(1,4)-mannan degradation | No specific species |
| P163-PWY | L-lysine fermentation to acetate and butanoate | *Fusobacterium hwasookii* |
|  |  | *Fusobacterium nucleatum ** |
|  |  | *Fusobacterium periodonticum* |
| P161-PWY | acetylene degradation (anaerobic) | *Fusobacterium nucleatum ** |
|  |  | *Olsenella uli* |
|  |  | *Streptococcus anginosus ** |
|  |  | *Streptococcus australis* |
|  |  | *Streptococcus gordonii* |
|  |  | *Streptococcus infantis ** |
|  |  | *Streptococcus oralis* |
|  |  | *Streptococcus parasanguinis* |
|  |  | *Streptococcus pneumoniae* |
|  |  | *Streptococcus sp F0442* |
| PWY-8131 | 5'-deoxyadenosine degradation II | *Catonella morbi* |
|  |  | *Lachnoanaerobaculum saburreum* |
|  |  | *Lachnospiraceae bacterium oral taxon 500* |
|  |  | *Olsenella profusa ** |
|  |  | *Pseudoramibacter alactolyticus* |
|  |  | *Treponema vincentii* |

Note: * for significant species.
